# Theory and application of an improved species richness estimator

**DOI:** 10.1098/rstb.2022.0187

**Published:** 2023-07-17

**Authors:** Eden W. Tekwa, Matthew A. Whalen, Patrick T. Martone, Mary I. O'Connor

**Affiliations:** ^1^ Department of Zoology, University of British Columbia, Vancouver, V6T 1Z4 British Columbia, Canada; ^2^ Department of Botany, University of British Columbia, Vancouver, V6T 1Z4 British Columbia, Canada; ^3^ Hakai Institute, Heriot Bay, V0P 1H0 British Columbia, Canada; ^4^ Department of Biology, McGill University, H3A 1B1 Montreal, Quebec, Canada; ^5^ Department of Biology, Virginia State University, Petersburg, 23806 VA, USA

**Keywords:** species richness, biodiversity, bias, occupancy, bootstrapping

## Abstract

Species richness is an essential biodiversity variable indicative of ecosystem states and rates of invasion, speciation and extinction both contemporarily and in fossil records. However, limited sampling effort and spatial aggregation of organisms mean that biodiversity surveys rarely observe every species in the survey area. Here we present a non-parametric, asymptotic and bias-minimized richness estimator, *Ω* by modelling how spatial abundance characteristics affect observation of species richness. Improved asymptotic estimators are critical when both absolute richness and difference detection are important. We conduct simulation tests and applied *Ω* to a tree census and a seaweed survey. *Ω* consistently outperforms other estimators in balancing bias, precision and difference detection accuracy. However, small difference detection is poor with any asymptotic estimator. An R-package, *Richness*, performs the proposed richness estimations along with other asymptotic estimators and bootstrapped precisions. Our results explain how natural and observer-induced variations affect species observation, how these factors can be used to correct observed richness using the estimator *Ω* on a variety of data, and why further improvements are critical for biodiversity assessments.

This article is part of the theme issue ‘Detecting and attributing the causes of biodiversity change: needs, gaps and solutions’.

## 1. Introduction

A central objective of biodiversity science is to understand how many species there are and how the number and kinds of species change across space and time [1]. Not only is species richness important for today's ecosystem and human health assessments [2–6], it is essential for inferring palaeoecological patterns and mass extinction events [7]. Estimating missing species or classes from limited observations is a fundamental problem in ecology [8–10], genomics [11], cryptography, machine learning and linguistics [12]. These examples show richness estimation has broad applications, yet current estimators have limited accuracy.

In biodiversity and conservation sciences, absolute richness estimation and detection of richness differences (spatial or temporal) are each challenging in their own ways [13–15] because of data limitation and statistical problems [16–21]. Observed species richness and detection of differences across communities or time are prone to data bias [22] and undocumented errors resulting from varying survey and sampling effort, design and observer skill [14]. These problems are more acute for species richness than for other biodiversity metrics [23], in part because richness weighs all species equally regardless of rarity. These data and statistical problems, along with other theoretical reasons, have led researchers to consider other biodiversity metrics focusing on abundance [23], community turnover [24], evenness-weighted diversity (Roswell *et al*. [25]) and interaction network (Chiu *et al*. [26]). However, richness remains a primary metric that is accessible to scientists and the public, is the main determinant of ecosystem functioning and stability in ecological theory [1], and is a measure of speciation and extinction [17]. Furthermore, knowing the number of missing species is critical to assessing how much sampling effort is required to accurately measure other biodiversity metrics (such as species interactions). Data-intensive methods such as multi-species occupancy models can estimate richness of targeted species groups, but they require repeated field surveys and are sensitive to models of abiotic and biotic drivers [27,28]. For many general applications and meta-analyses, it is desirable to have an asymptotic (finite projection at infinite sampling effort) richness estimator that can both correct bias (uncover true absolute richness) and accurately detect spatial and temporal differences across communities.

Commonly, biodiversity observations include individual counts identified to the species level. One obvious way to improve biodiversity observation is to increase sampling effort [29], but this is costly and not possible for some taxa, habitats and historical samples. Insufficient sampling effort can increase bias, reduce estimation precision (repeatability) and undermine efforts to detect change over time, but a robust, unbiased estimator can recover missing observations to some extent. The most robust asymptotic richness estimator that uses abundance data remains the Chao1 method [8,30]. Chao1 and related estimators are based on Turing's sampling theory that relates the number of unobserved classes (codes or species) to observed rarity [30,31]. These methods are expected to be inaccurate with spatial heterogeneity and heterogeneous observation probabilities [32]. Other estimators rely on tuning parameters, but these methods can be unstable and are still under development [33]. There is a need to develop new statistical methods that fully use information in common biodiversity survey design datasets, without having to separately estimate additional quantities that may be harder to obtain than richness itself [27].

**Figure 1.**
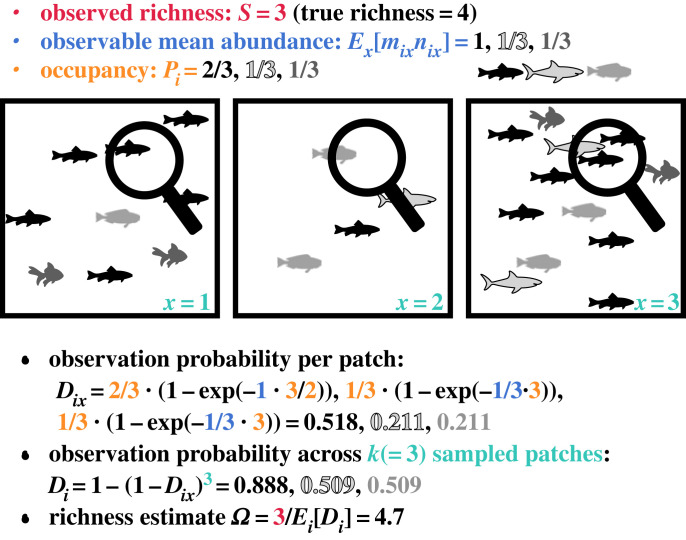
From spatial statistics to richness estimate. Spatial statistics are used to infer observation probabilities, leading to a bias-corrected richness estimate *Ω*. Statistics for the three observed species (black fish, outlined grey shark and grey fish) are observable mean abundance *mn* (blue: three black fish, one grey shark and one grey fish across three patches), occupancy *P* (orange: two-thirds by black fish and one-third occupied by each of grey shark and grey fish) and sites sampled *k* (cyan: three). Observation probability per patch and across patches are defined in equations (2.2) and (2.3), and *Ω* is defined in equation (2.5).

Here we outline four technical problems that an ideal asymptotic estimator should solve. The first two are human-induced biases to species observation: the fraction of individuals observed and the number of patches (sites, transects or quadrats) sampled are expected to influence observed richness. The next two are natural properties of biological distributions that can also influence observation: abundance and patch occupancy (with effects foreshadowed in figure 1). These variables will be precisely defined, and we use them to mechanistically derive observation probabilities and a set of what we will call *Ω* bias-corrected asymptotic richness estimators. This approach departs from both Turing's reliance on rarity, which is prone to noisy observations, and from earlier parametric extrapolations that have weak justifications [30]. Using the derivations, we highlight how means and variances of the variables affect richness observations. Next, we evaluate the performance of three proposed *Ω*-estimators relative to six other established asymptotic estimators (Chao1, Gamma-Poisson, Chao2, abundance-based coverage estimator (ACE), Jackknife1-abundance, Jackknife2-incidence [8,30,34–37]) using simulations designed to challenge each estimator. We use six performance measures to evaluate bias (under- or over-estimation tendency), precision (repeatability of estimates) and accuracy (error magnitude and spatial/temporal trend detection). We additionally devise a bootstrapping procedure that accounts for the spatial and species dependence to quantify precision from a single survey. To test our method with empirical applications, we use two multi-year, multi-patch datasets: the Barro Colorado Island tree census [38], which probably reflected true richness and a negative temporal trend, and the British Columbia intertidal seaweed survey [39], which probably was incomplete with an unclear temporal trend. Even when true richness is unknown, spatial subsampling and local downsampling experiments on the real datasets allow us to explore estimators' consistency across real conditions. Finally, we provide Matlab codes [40] and an R-package*, Richness*, which generate a suite of asymptotic richness estimates (https://github.com/EWTekwa/Richness).

## 2. Methods

The general reader aiming to understand the performance and application of the proposed estimator may skip the following *Richness estimator derivation* and *Simulation set-up* subsections on first reading, and instead focus on evaluation and application sections that follow. The technical subsections will be of greatest interest to statisticians, but would also aid the general reader in understanding why our proposal works. The data and code used in this paper are available on a Zenodo repository at [40]. The R-package *Richness* is available at https://github.com/EWTekwa/Richness, which contains the main function *EstimateRichness* that takes in spatial abundance data and returns point estimates and bootstraps (see next section) for observed richness, Chao1, Gamma-Poisson, Chao2, ACE, Jackknife-abundance, Jackknife-incidence, *Ω*, *Ω_T_* and *Ω*_0_.

### (a) Richness estimator derivation

Here we define variables that can be measured from a spatially partitioned abundance dataset, with the aim of arriving at a bias-corrected richness estimator. Our strategy involves the following steps (figure 1). We define ‘patch’ *x* as the spatial unit where individuals can be counted, and a community consists of a collection of *N* patches (including those sampled and unsampled). We model how both spatial heterogeneities across patches and observation errors within patches affect the probability that a species is observed at least once in the community. We then use spatial abundance statistics of observed species to estimate true richness, which is the observed richness divided by some estimated observation probability.

Each species *i* has a true mean density *n_i_*, which is the total number of individuals in the community divided by the true number of patches *N*, or equivalently the individuals per patch *x* across all true patches (*n_i_* = *E_x_*[*n_x_*]). The fraction of individuals observed at *x*, *m_ix_*, is also understood as the individual catchability of species *i* [35]. Thus, *m_ix_n_ix_*, simplified as (*mn*)*_ix_*, is the local ‘observable abundance’. The set of species *i*'s observable abundances averaged across space, (*mn*)*_i_*, will serve as the first (non-spatial) random state variable in our subsequently developed richness estimator.

In contrast with the above community scale, let us descend to the local patch scale where observation occurs as a function of within-patch density (*n_ix_*). Specifically, local observation probability (*D_x_*_|_*_nix >_*_0_) is a function of *n_ix_* within occupied patches (*n_ix_*_|_*_nix >_*_0_). The mean within-patch density of occupied patches *n_i_*_|_*_nix >_*_0_, is $\sum_{x|n_{ix}\rangle 0}^{NP_{i}\;}n_{ix}/(NP_{i})$ where *p_i_* is occupancy, or the fraction of patches where species *i* is present. On the other hand, mean density *n_i_* is $\sum_{x}^{N}n_{ix}/N$. Since in unocccupied patches *n_ix_*= 0, *n_i_* can be written as $\sum_{x|n_{ix}\rangle 0}^{NP_{i}}n_{ix}/N$. Thus, *n_i_*_|_*_ni>_*_0_ *= n_i_*/*p_i_*. When a fraction *m* of individuals are observed, on average there are (*mn*)*_i_*/*p_i_* observable individuals in occupied patches. Using this mean, the local observation process can be described by a non-spatial Poisson sampling process (sampling with replacement). This local Poisson sampling process should not be confused with a Poisson spatial distribution across patches, which was not assumed. Sampling with replacement is likely in non-destructive field surveys when individuals move and their identities are not tracked; on the other hand, sampling without replacement approaches Poisson sampling when *m* is small. When *m* approaches 1, observation probability for sampling without replacement will be underestimated by Poisson (overestimates richness as will be seen by inspecting equation (2.4)), but correction hinges on knowing *m* [41], which we assume to be unavailable. We write the local observation probability within occupied patches as 1 minus the rate of failure to observe:

2.1 $$D_{ix|n_{ix}>0}=1-\exp\left(-\frac{{\left(mn\right)}_{i}}{P_{i}}\right).$$

Now we zoom back out to the community level and consider spatial processes across multiple patches that may be occupied or not. The observation probability of species *i* per random patch *x* is the occupancy times the conditional local observation probability (equation (2.1)) when the patch is occupied:

2.2 $$D_{ix}=P_{i}\left(1-\exp\left(-\frac{{\left(mn\right)}_{i}}{P_{i}}\right)\right).$$

The observation probability of species *i* within the community is 1 minus the cumulative probability of failure to observe, the latter being the probability of failure to observe within patch raised to the power *k_i_* (our fourth state variable) if patches are randomly picked with replacement. The result does not differ from picking without replacement when *k_i_* is much smaller than the true number of patches. Otherwise, sampling with replacement reduces the chance of observing the same unoccupied patch, so in this situation assuming sampling without replacement underestimates observation probability (overestimates richness). The sampling with replacement observation probability across the community (*D_i_*) is

2.3 $$D_{i}=1-{\left(1-D_{ix}\right)}^{k_{i}}.$$

If patches are not randomly picked, then *k_i_* should be the effective sample size. As an extreme example, when only one patch is repeatedly sampled at the same time in exactly the same way, effectively *k_i_* = 1. In practice however, even sampling the same patch repeatedly would generate different individual and species counts when catchability *m* is below 1 and/or individuals move between patches during the finite time separating samples. Ignoring the non-independence of spatial samples would inflate *D_i_* (underestimates richness), but defining what is meant by effective sample size will require nuanced considerations of how the spatial sampling design relates to the community's spatial distribution. We will assume *k_i_* samples are independent and revisit sample non-independence in the Discussion.

The observed richness *S* can be written without additional assumptions as the true richness *Ω* multiplied by the expectation of *D_i_* across truly present species *i* as a function of, ***Φ*** which includes *R* state variables *ϕ_r_*. In our observation process model, *R* = 3 and *ϕ_1_* to *ϕ_3_* are (*mn*)*_i_*, *P_i_* and *k_i_*:

2.4 $$S=\Omega E_{i}[D_{i}(\Phi )].$$

By rearranging equation (2.4), we define *Ω* (equation (2.5)) as our proposed ‘exact’ bias-corrected richness estimator using the average observation probabilities based on state variables $\hat{\;\Phi }$ measured from the set of observed (rather than truly present) species (see figure 1 for numerical example). All quantities with a $\hat{\mathrm{hat}}$ denote observed rather than true values. The estimator *Ω* contains survivorship bias [42]—a type of sampling bias stemming from the difference between $\hat{\;\Phi }$ and ***Φ***, because $\hat{\;\Phi }$ is only measurable for observed species. For intuition, observed species (those that ‘survived’ sampling) will probably have higher abundances than the average of truly present species because rare species tend to be missed, so the mean observed abundance is higher than the true mean abundance of all species:

2.5 $$\Omega \approx \frac{S}{E_{i}[D_{i}(\hat{\;\Phi })]}.$$

An alternative is to write $E_{i}[D_{i}(\Phi )]$ in equation (2.4) in terms of a second-order Taylor expansion (with *r* and *q* being variable indices), which provides theoretical insights into how means and variances of different statistics affect the observation process. The Taylor estimator *Ω_T_* is

2.6 $$\;\Omega_{T}\approx \frac{S}{D_{i}(E_{i}[\hat{\phi }])+\frac{d^{2}D_{iM}(E_{i}[\hat{\phi }])}{2d{\left(mn\right)}^{2}}{\mathrm{var}}_{i}(\hat{{\left(mn\right)}_{i}})+\frac{d^{2}D_{iM}(E_{i}[\hat{\phi }])}{2dP^{2}}{\mathrm{var}}_{i}(\hat{P_{i}})+\frac{d^{2}D_{iM}(E_{i}[\hat{\phi }])}{d(mn)dP}{\mathrm{cov}}_{i}(\hat{{\left(mn\right)}_{i}},\hat{P_{i}})}.$$

$E_{i}[\hat{\phi }]$ stands for the average values of the state variables across observed species. The derivatives in the denominator can be tracked as the coefficients *c_1_*, *c_2_* and *c_3_*. *k* is assumed to have no variance since spatial surveys commonly aim to discover all possible species at the same time, hence there is not a second-order correction term for *k* (but can be easily included otherwise). Higher moments are increasingly harder to estimate from a limited number of observed species and may destabilize the estimator. We chose to truncate *Ω_T_* at the second statistical moments, but we can even ignore the second moments. The resulting estimator, *Ω*_0_, keeps only the first term of the denominator in equation (2.6):

2.7 $$\;\Omega_{0}\approx \frac{S}{D_{i}(\hat{\phi })}.$$

The estimators *Ω*, *Ω_T_* and *Ω*_0_ have idealized counterparts that eliminate the survivorship biases in the observed means, variances and covariances of *mn* and *P*. This is achieved by taking out $\hat{\mathrm{hat}}$ in equations (2.5)–(2.7); let us call the resulting corrected estimators *Ω_C_*, *Ω_TC_* and *Ω*_0_*_C_*. These corrected estimators can only be used in simulations where the state variables are known. Note we kept *S* as the observed richness, which clearly contains survivorship bias, but the biases in the correction terms were eliminated. Thus, the performance of these corrected estimators represents how much *Ω*, *Ω_T_* and *Ω*_0_ can improve in the future.

### (b) Quantifying precision by bootstrapping

From a single survey, we produce approximations for the standard deviation (s.d.) interval and the coefficients of variation (CV = variance/mean) of each estimator from bootstraps. These quantify the precision, uncertainty or repeatability of estimates (small s.d. and CV mean high precision). We used non-parametric bootstrapping [43] because the distribution of possible estimates for a single community is *a priori* unknown.

A key consideration in our implementation of bootstrapping is that individual occurrences are not independent both within species and patches because of the intraspecific tendency to cluster [44,45]. By contrast, common bootstrapping procedures for richness estimates assumed that sampled individuals or sample sites are independent (as did the estimators themselves) [8], an assumption that is also common in rarefaction and extrapolation techniques that control for sampling effort or coverage when comparing communities [14] but can lead to erroneous uncertainty estimates. To account for spatial and species dependencies of sampled individuals, we follow a simple block bootstrapping procedure [46]. For each bootstrap, we first randomly sampled with replacement *k* transects (electronic supplementary material, figure S4). Then, from this spatially randomized set, we randomly sampled with replacement as many species as the point estimator predicts from the original dataset (e.g. *Ω* or Chao1). All species were drawn from the observed set of *S* species, but the bootstrap pseudocommunity now consists of a potentially larger number of pseudospecies (see [8]). We used this bootstrap sample size instead of the observed *S*, because we know *S* underestimates the true community richness and bootstrapping is intended to recreate sampling variations from that community. s.d. and CV were computed from the bootstrap estimates. These measures allow us to measure an estimator's precision independently from bias.

### (c) Simulation set-up

To test and compare the performance of asymptotic richness estimators, we simulated spatially partitioned communities under four scenarios that present different challenges that arise from imperfect local observation and spatial heterogeneities. For each simulation scenario, we created 2000 unique communities. Each community has 100 patches so that it is possible to sample a small fraction, and 1 to 100 species are present, which represents a common range for many focal taxa (such as what was observed in the seaweed dataset used later). Parameter values vary between communities within a scenario but converge on scenario mean values in abundance *E*[*n*] and occupancy *E*[*P*], which simulate natural variations in spatial biodiversity patterns that we may want to discern. Further, each community was sampled at different efforts, converging on mean spatial sampling effort (patches sampled *E*[*k*]) and fraction of individuals observed *E*[*m*]. Thus, each of the 2000 communities represent a unique set of biological and sampling conditions.

Specifically, each simulated community's mean fraction of individuals observed $\tilde{m}$ was drawn from *E*[*m*] + (*U*(0,1)−0.5)*E*[*m*](1−*E*[*m*]) where *U* is a uniform random value. Each species in a community in turn had a *m_i_* value drawn from the same formula above but with E[*m*] replaced by $\tilde{m}$. The community mean occupancy $\tilde{P}$ was drawn from *E*[*P*] + (*U*(0,1)−0.5)*E*[*P*](1−*E*[*P*]), and each species has a *p_i_* value drawn from the same formula but with E[*P*] replaced by $\tilde{P}$. Each community's mean abundance $\tilde{n}$ was drawn from 2E[*n*]*U*(0,1) and species *n_i_* was obtained from the following steps. First, a number $\overset{\prime }{\underset{i}{n}}$ was drawn from a lognormal distribution [47] with a mean of ln($\tilde{n}$)−0.5 an s.d. of 1, all divided by $\tilde{P}$. Then, all species with $\overset{\prime }{\underset{i}{n}}$ less than 1 was assigned the value of 1, and lastly $n_{i}=\overset{\prime }{\underset{i}{n}}\left(\sum {n^{\prime }}_{i}+\sum_{{n^{\prime }}_{i}<1}{n^{\prime }}_{i}-\sum_{{n^{\prime }}_{i}<1}1\right)/\sum \overset{\prime }{\underset{i}{n}}$ to ensure both the desired $\tilde{n}$ and $\tilde{P}$ are achieved by the following individual assignments (electronic supplementary material, figure S1*e*). We assigned individuals to each patch by drawing from a Poisson distribution with the mean being *n_i_* multiplied by 1 if drawn from *U*(0,1)<*P_i_* and 0 otherwise. Finally, subsampling biodiversity surveys were simulated by randomly picking *k*∊ patches in each community (without replacement). Within each patch and for each species, individuals were observed by drawing from a Poisson distribution (sample with replacement) with the mean being (*mn*)*_i_*.

**Figure 2.**
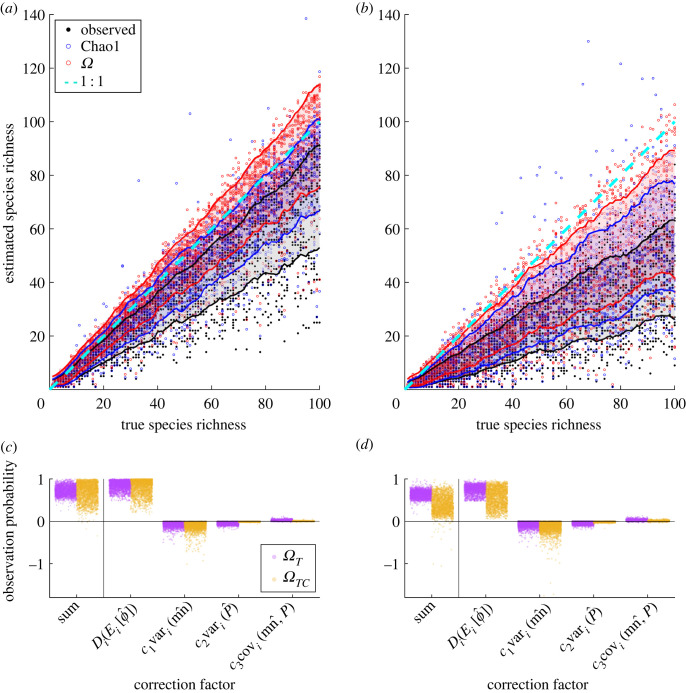
Simulated richness estimates under mixed conditions. Two thousand communities are populated with 1–100 species over 100 spatial patches, and 2–10 patches (*k*) are sampled for richness estimates, including Chao1 (blue circles), *Ω* (red circles) and observed (black dots). Blue, red and black shades and lines are s.d. bounds for the estimators, and the dotted cyan line is the 1 : 1 reference. (*a*) Imperfect local observation and spatial heterogeneity scenario (figure 2*c*). (*b*) Poor local observation and spatial heterogeneity scenario (figure 2*d*). (*c,d*) Terms contributing to estimated cross-species observation probability by *Ω_T_* (purple, equation (2.6)) and the corresponding idealized *Ω_TC_* where spatial abundance statistics are corrected for survivorship bias (yellow). Sum is the final estimated observation probability.

### (d) Performance evaluation

We desire asymptotic richness estimators to be precise (repeatable), unbiased and accurate, but these components may exhibit trade-offs given limited information [48]. Thus, the performance of an estimator cannot be summarized by a single measure; we present six measures to assess whether certain estimators rank higher in one aspect and lower in others.

A least-squares linear regression corrected for heteroscedasticity [49] (*hac* function in Matlab R2020a) was performed on the estimated richness versus true richness within each estimator to address richness-dependent bias and precision. Measure no. 1 is the regression slope. A slope less than 1 indicates that more diverse communities are more severely underestimated than less diverse communities, which is a prevalent bias among richness estimators (sometimes expressed as 1 minus slope [48]). We report the s.d. in the regression slope (measure no. 2) as a measure of the estimator's precision around a bias. A low s.d. indicates the estimator is precise (consistent) across communities and true richness. In addition, R^2*^ was computed for the 1 : 1 line, which describes the deviation from true richness (measure no. 3). A small R^2*^ indicates either underestimates or overestimates, with large errors capable of generating negative values (worse than simple average). R^2*^ is an overall measure of the point estimate's accuracy. R^2*^ contrasts from the *R^2^* of the true : estimate linear regression, the latter having been used to measure precision [48] but we believe the measure is confounded with bias. Even if an estimator always returns the same richness value for a given true richness (high precision), bias would reduce *R^2^* below 1, thus we chose to use slope and s.d. in slope as our measures of bias and precision. Richness difference detections (correct sign of difference) were checked between 2000 randomly picked community pairs that were different in richness by at most 2, 10 or 20 but were not identical (measure nos. 4–6). Detection accuracy is essential to infer spatial or temporal trends, which are major objectives of biodiversity studies. Detection accuracies above 0.5 is better than a coin-flip, since the random outcomes are whether community A has a higher or lower richness than community B, with equal probabilities of each being true across the 2000 comparisons.

For each of the six performance measures above, we computed an estimator's performance deviation from the mean across 13 estimators, then divided by the s.d. to obtain a score. Performances for measure nos. 4–6 are just as defined above. For measure no. 1, performance was 1-|slope-1|; for measure no. 2, performance was 1−R^2*^, ensuring that all large performance scores are better. The scores were averaged across the six metrics, with 1 and −1 corresponding to performances that are one s.d. better or worse than the average estimator.

To visualize estimations, we computed the mean and s.d. in estimates that occur in a rolling window of 10 centred around each true richness. The s.d. bounds are plotted around the means. We then compared these bounds with the same rolling window averages of within-community bootstrapped s.d. estimates across true richness. The s.d. bounds estimated from 50 bootstraps of a single community should ideally estimate the s.d. across multiple communities with the same true richness. Thus, when the two sets of bounds agree, the bootstrapping procedure is supported. These bounds are also linked to the slope s.d. introduced above as measures of precision: imprecise estimates should lead to a high slope s.d. independent of bias.

**Figure 3.**
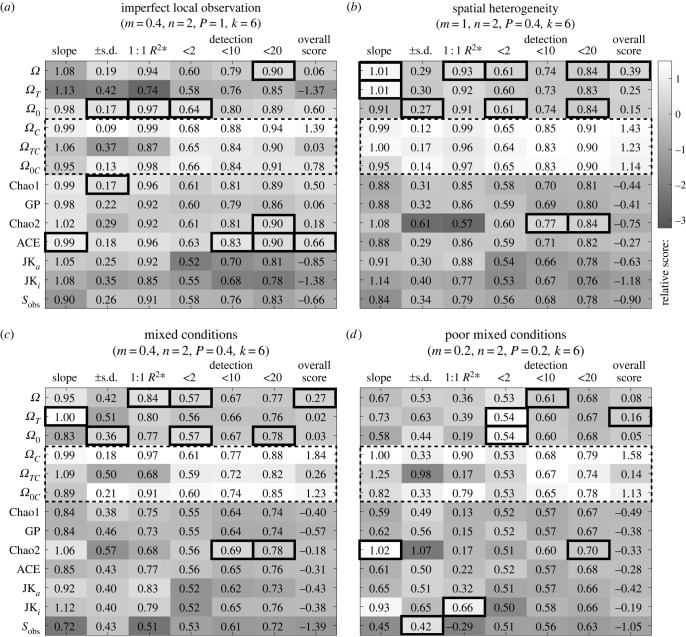
Simulation results. Estimator performances across four scenarios (*a*–*d*) are measured as the slope of estimated richness when regressed on true richness (downward bias when less than 1), s.d. in regression slope (precision), *R*^2*^ of the 1 : 1 line (overall accuracy) and pairwise difference detection accuracy when randomly picked communities were within a richness difference of 2, 10 or 20 (detection accuracy). Scores (shades—see legend) show how many s.d. an estimator outperforms (+) or underperforms (−) the averages of all estimators. Boxes highlight top scores excluding idealized estimators (dotted outline). Overall score is the average of six scores. Displayed fractions of individuals observed (*m*), abundance (*n*), occupancy (*P*) and sites sampled (*k*) are averages across species and community variations within the scenario.

We observed from simulations that *Ω_T_* and *Ω_TC_*'s estimated observation probabilities can occasionally be very small or negative because *mn* variance in particular can be numerically unstable (overestimated) from limited sample size (number of observed species). We set a threshold of 10% estimated observation probability below which *Ω_T_* and *Ω_TC_* switched to *Ω*_0_, which cannot be negative and is numerically stable.

### (e) Empirical datasets

The first dataset that we will use to test estimators is a high-quality Barro Colorado Island tree census from Panama [38], which exhibits a negative temporal trend that is probably true [50]. Starting in 1982, every stem was identified by species every 5 years, for a total of eight census. Over 300 species were observed across 1250 quadrats fully covering a rectangle over 50 hectares.

The second dataset is an extensive seaweed diversity survey collected annually from 2012 to 2019 on the coast of British Columbia, Canada. The dataset features relatively high standards in sampling effort and taxonomic identification for the marine realm [39] but is not a census. The survey tracked seaweed species and their abundance (estimated as per cent cover). Nine transects were established. Each year, 10 quadrats were randomly selected on each transect and surveyed for per cent cover. Between 73 and 82 species were observed each year. Transects probably represent independent spatial samples of an underlying community, whereas quadrats were less spatially independent, so we aggregated quadrat data within transects for this analysis. A 10 × 10 grid was overlaid on the 0.5 m × 0.5 m quadrat, and any organism filling each square of this grid added 1% to the species’ total cover within the quadrat [51]. A species that was partially present in only one square of the grid was assigned a per cent cover of 0.5 (or trace). Since 0.5 is the lowest recorded cover and is akin to one individual count being the minimum, we divided all per cent cover data by 0.5 to obtain abundance.

Abundance within the context of sampling has no real biological meaning beyond being discrete; the abundance unit does not have to coincide with the individual organism, a concept that we note is open to biological debates and subject to evolution [52,53], but has no consequences for richness estimation. The only important feature of count data is that the observable abundance *mn*, not the biological *n*, approximates a Poisson process; *p* is not affected by a change of unit and is thus insensitive to the individual definition. The other consideration for converting per cent cover to observed local abundance (*mn*) is that it is capped and thus not quite Poisson, but in practice recording abundances beyond this cap will probably not affect the estimated observation probability (e.g. failure to observe, exp(-*mn/p*), is 4 × 10^−44^ for *mn/p* = 100 in equation (2.1)).

In addition to obtaining point estimates of richness on both full datasets, we subsampled transects and quadrats in different combinations and produced replicated subsample estimates. For each subsampling replicate for the Barro Colorado Island tree census, we randomly picked a subset of quadrats (patches) without replacement and sampled these same quadrats every year. For each subsampling replicate for the British Columbia seaweed survey, we randomly picked transects and quadrats (without replacement) every year. Quadrat counts were summed to obtain transect data, so transects were treated as patches here. Each spatial effort level also received a complementary local downsampling experiment in which only 10% (*m* = 0.1) of individuals in each selected transect was observed. We call this locally imperfect observation experiment ‘downsampling’ as opposed to ‘subsampling’ to distinguish it from spatial subsampling. For each experiment, we replicated the subsampling 40 times since different transects and quadrats could be randomly picked. An ideal estimator would predict the same true richness regardless of spatial subsampling and local downsampling.

The results were presented through five metrics. First, the point estimates for Chao1, Chao2, *Ω* and observed richness were plotted against subsample experiments in two groups, which are the experiments without and with local downsampling. Second, the CV were plotted to measure precision (see *Quantifying precision by bootstrapping* section). CV was obtained from either 2000 bootstraps of the full dataset, or 50 bootstraps in each of 40 subsampled and downsampled experiments that were then averaged across experiments. Third, we plotted the subsampled and downsampled richness estimates as the portion of richness estimates obtained from the full datasets (% of full estimates), which create a multi-dimensional rarefaction (with 100% indicating asymptote is reached). These relative measures show how close are the estimators to asymptotes with lowered efforts. Fourth, we performed linear regressions of point estimates on year or census (time) and plotted the slopes as the detected temporal trends. Fifth, we plotted the *p*-value of the slope being different from the null of no trends, which should approach zero if a true trend exists (likely for Barro Colorado Island trees) and larger if no trends exist (likely for British Columbia seaweeds).

**Figure 4.**
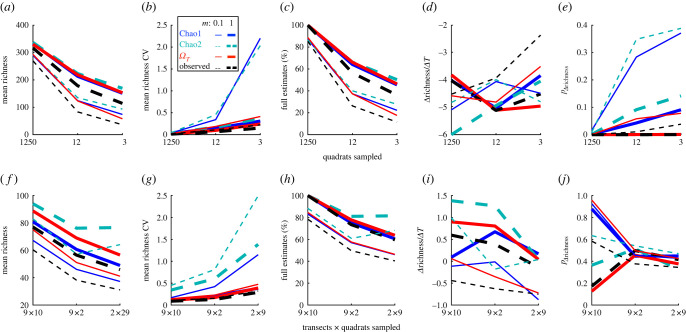
Richness estimates in application. (*a–e*) Barro Colorado Island tree census. (*f–j*) British Columbia seaweed survey. Results for the four richness estimates averaged across years are plotted for different spatial subsampling experiments (*x*-axis), with local individual downsampling scenarios (portion of individuals observed from full dataset *m*) indicated by line thickness (see legend in (*b*)). (*a,f*) Point richness estimates. (*b,g*) Richness coefficients of variation (from all bootstraps and subsampled replicates) averaged across years. (*c,h*) Percentages of full estimates as a function of spatial subsampling (rarefaction curves—note for seaweed survey spatial subsampling is categorical and not strictly ordered), which is 100% when asymptote is reached. (*d,i*) Detected temporal richness changes (with *Δ*T being a census or a year) from slope of linear regressions of point estimates averaged across bootstraps and subsamples on time. (*e,j*) *p*-values for detected temporal changes (in (*d,i*)) being different from zero.

For each full dataset, we plotted the s.d. from bootstraps in each year. For subsampling experiments, we plotted the s.d. across subsamples in each year. Both precision measures represent observation variations expected from repeatedly sampling the true community.

## 3. Results

### (a) Theoretical insights

Here we describe how sampled patches (*k*) and means and variances in observable abundance (*mn*) and occupancy (*p*) affect species observation and induce bias to the observed richness. First, we found that the effects of means of these variables (across species) were captured by *D*_*i*_ in the richness estimator *Ω*_0_ (equation (2.7)), which is the single-species observation probability (equation (2.3)) computed at variables’ mean values (as if all species in the community share the same characteristics). Equations (2.2) and (2.3) show that observation probability increases with means of *k, n*, *m*, and *p*, results that can also be visualized in figure 1.

Across species variance effects (of *mn* and *p*) depend on the signs of the second-order partial derivatives according to the Taylor expansion in the estimator *Ω_T_* (equation (2.6)). The analytical expressions are long but always negative (see numerical outcomes in figure 2*c,d*). Therefore, variances always decrease the overall observation probability. The effect of the covariance between *mn* and *p* can be either positive or negative. Precise numerical effects are outputs of the function *estimateRichness* in the R-package *Richness* when applied to user's spatial abundance data.

These results clarify two main ways in which non-richness biodiversity components induce bias to observed richness. Occupancy is related to spatial turnover and dissimilarity, yet its mean value affects species observation (equation (2.3)). Variance in observable abundance, which is probably inversely proportional to community evenness (assuming *m* is not anti-correlated with *n*), reduces observed richness even though true richness should not be affected by evenness. These effects illustrate the need for biodiversity metrics to be bias-corrected if they are to represent their definitions.

### (b) Simulation tests

We tested the proposed estimators (*Ω*: equation (2.5), *Ω_T_*: equation (2.6), *Ω*_0_: equation (2.7)) on a simulated dataset of species abundance. We compared these to other leading estimators including: the unbiased Chao1 [8,30], the Gamma-Poisson correction of Chao1 (GP) [34], Chao2 [35], ACE [36], Jackknife for abundance data (JK_a_) and Jackknife for incidence data (JK_i_) [37]. We also tested the idealized versions *Ω_C_*, *Ω_TC_* and *Ω*_0*C*_ that are only operational in simulations where the true spatial statistics are known for all species to establish theoretical limits of *Ω*-type estimators. For incidence-based estimators including Chao2 and Jackknife2, abundance within patches were converted to presence/absence. The six performance statistics in figure 3 are described in the Methods: *Performance evaluation* section. The electronic supplementary material, figure S3*a,b* correspond to scenarios I and II in figure 3, and figure 2*a,b* correspond to scenarios III and IV. These figures show s.d. bounds for observed richness, Chao1 and *Ω* across communities sharing similar true richness. The electronic supplementary material, figure S2 shows that s.d. bounds from bootstrapping within each community are similar to the across-community s.d. bounds across scenarios, which supports the bootstrapping procedure for quantifying precision.

The simulations show that our proposed *Ω* and *Ω*_0_ estimators were the only non-idealized estimators that performed above average across all imperfect observation and spatial heterogeneity scenarios according to the overall score that summarizes bias (true : estimated richness slope), precision (slope s.d.), accuracy (*R*^2*^) and difference detection accuracy (detection; figure 3). *Ω* performed better than *Ω*_0_ in all but scenario I where true occupancy was one for all species. *Ω*'s bias was lower than other abundance-based estimators but higher than incidence-based estimators in some scenarios, although these incidence-based estimators also had the lowest precision accompanied by low overall accuracy and/or low detection ability (figure 3). All estimators performed better than no corrections (observed richness) except for Jackknife estimators in some scenarios (figure 3*a*,*b* overall scores). *Ω* and *Ω*_0_ had higher precision when compared to observed richness except under the poorest conditions (figure 3*d*). Under the poorest scenario (figure 2*b*), only *Ω*-type estimators were better than coin flips in detecting richness differences that were less than two (figure 3*d*), although these small differences were challenging for any estimator (even idealized ones) to detect given the small spatial sampling effort (*k*).

We found that *Ω_T_* and the idealized *Ω_TC_* outperformed existing estimators when spatial heterogeneity was present (figure 3*b*–*d*). However, they were less stable than *Ω* and *Ω*_0_, particularly in terms of accuracy and performance variation across scenarios, indicating that the variances and covariances in the Taylor expansion are numerically difficult to estimate. The proposed *Ω* and *Ω*_0_ gained 2–7% in detection accuracy compared to using observed richness across scenarios. The idealized version of *Ω* (*Ω_C_*, only operational in simulations) outperformed all others by all measures and scenarios, further improving difference detection accuracy (up to 17% higher than observed) while eliminating bias, increasing precision, and achieving 90% overall accuracy even under the poorest conditions (figure 3).

The room for improvement to *Ω*-type estimators comes from survivorship bias in observed *mn* and *p*. This bias can be seen in the differences between the *Ω_T_* and *Ω_TC_* correction factors in the imperfect detection versus spatial heterogeneity scenarios (figure 3*a*,*b*; electronic supplementary material, figure S3*c,d*), which are also relevant for *Ω* because the Taylor expansion version is an approximation of *Ω*. Under imperfect local observation, observed mean *mn* was biased upwards and variance downwards because rare species were missed (electronic supplementary material, figure S1*a*). The upward bias in mean *mn* overestimated observation probability while downward bias in variance had the opposite effect according to the effects derived in the *Theoretical insights* section. Mean observed *p* was biased downwards and variance was biased upwards (electronic supplementary material, figure S1*b*). The downward bias from mean *p* and upward bias from variance in *p* led to underestimates in observation probability (upward bias, electronic supplementary material, figure S3*a,c*). For the scenario with spatial heterogeneity, the biases in variance of *mn* and mean of *p* were the opposite of those caused by imperfect local observation (electronic supplementary material, figures S1*d* and S3*b,d*). The covariance correction factor was relatively small and similar in *Ω_T_* and *Ω_TC_* (figure 2*c,d*). The opposing effects of biases across variables and sources partly cancelled each other, reducing net bias in the *Ω* and *Ω_T_* richness estimates to various degrees (figure 2). In comparison, other estimators depended only on either abundance or spatial occupancy biases and therefore cannot cancel each other [8]. When the variables (inaccessible in real datasets) were unbiased, overall bias was reduced or eliminated (*Ω_C_* in figure 3). Consistent directions in the survivorship biases associated with some biotic and abiotic sources suggest that corrections are possible to some extent; we identify these as future research objectives. The above insights also apply to *Ω*_0_ but only through the opposing biases in means of *mn* and *p* under imperfect observation, and their aligning biases under spatial heterogeneity. The consequence is that when little (biological and observational) spatial heterogeneity is present, *Ω*_0_ outperforms *Ω* (figure 3*a*); otherwise *Ω* is superior. We will focus on applying *Ω* to datasets.

### (c) Barro Colorado island tree census

We first computed the observed richness (all species recorded), Chao1, Chao2 and the proposed *Ω* point estimates as well as bootstrapped s.d. bounds for each year using the full census data (electronic supplementary material, figure S5*a*). Observed spatial statistics are shown in the electronic supplementary material, figure S6. All estimators predict richness close to the observed richness (figure 4*a*), suggesting the census was accurate. All estimators showed similar bootstrapped precision as measured by the CV (figure 4*b*). Based on the observed data, a negative trend of four species lost per census (per 5 years) was observed (figure 4*d*; average *p* = 2 × 10^−5^ from bootstrap least-squares linear regression). This trend is probably reliable owing to the high-quality census. All estimators agreed with a negative trend with low *p*-values.

Next we examined how the estimators perform when only 12 or 3 quadrats out of 12 50 were subsampled through the census years. Across 40 replications for each of the spatial subsampling experiments, the observed richness and *Ω* estimates at 12 sampled quadrats continued to indicate strong evidence for a negative trend (figure 4*d,e*), while Chao1 and Chao2 showed less confidence in such a trend (figure 4*e*). When spatial subsampling was coupled with local downsampling of individuals observed, precision decreased much more for Chao1 and Chao2 compared to *Ω* (figure 4*b*), particularly for Chao2 (electronic supplementary material, figure S5*f*). Low precisions probably contributed to the decreased ability to detect a temporal trend (if the trend from the full dataset is true). *Ω*'s correction was probably suppressed by the non-independence of quadrat samples (effective *k_i_* in equation (2.3) is probably smaller than the number of quadrats sampled). In the downsampling experiments where *m =* 10% of individuals were recorded, observed richness continued to detect a temporal trend (figure 4*d,e*), and *Ω* came much closer than Chao1 and Chao2 to detecting the same trend at 12 and 3 sampled quadrats (*p* = 0.06, 0.07 in figure 4*e*).

Overall, *Ω* exhibited similar downward bias compared to other estimators (including observed richness) under all subsampling and downsampling, but was less biased than observed richness (figure 4*a,b*). The results are consistent with the simulation analyses, which revealed *Ω* is most likely to detect richness differences, and Chao2 should be the least precise (figure 3).

### (d) British Columbia seaweed survey

In this marine seaweed diversity dataset, all estimates based on the full dataset indicated that richness is higher than observed (figure 4*f*; electronic supplementary material, figure S7). *Ω*_,_ Chao1 and Chao2 indicated that about 4, 12 or 17 species remained unobserved, respectively. Chao2 estimates were particularly uneven and imprecise across years (electronic supplementary material, figure S7; figure 4*g*). Observed spatial abundance statistics are shown in the electronic supplementary material, figure S8. The observed richness across years indicated a weak signal of temporal increase but was inconclusive (*p* = 0.17), while *Ω* indicated slightly more support for a positive trend (*p =* 0.13*;* figure 4*i,j*). Chao1 and Chao2 also indicated positive temporal trends but with much higher *p*-values. Given these statistics, the true temporal trend was probably weak but possibly positive (figure 4*i*). When the full spatial sample was locally downsampled (*m =* 0.1), Chao2 was closest to the observed richness followed by *Ω* (figure 4*f*), and all estimators and observed richness indicated no temporal trends (figure 4*j*).

Under the spatial subsampling experiment of nine transects with two quadrats each and either with or without local downsampling, Chao2 was the closest to the observed richness in the full dataset (presumably least biased; figure 4*f*) but the least precise (figure 4*g*). All estimators showed no evidence of temporal trends (figure 4*j*).

Overall, in this dataset *Ω* was the most precise and less biased than Chao1 under subsampling and downsampling relative to the observed richness in the full dataset (figure 4*f,h*), while Chao2 was the least biased but had the lowest precision. These results are consistent with the simulation analyses, with Chao2 and *Ω* being less biased than Chao1, but Chao2 being the least precise (figure 3).

## 4. Discussion

We derived a new set of *Ω* richness estimators (*Ω*: equation (2.5), *Ω_T_*: equation (2.6), *Ω*_0_: equation (2.7)) and used simulations and real applications to show they approach true richness and detect richness differences when the data are imperfect, spatially incomplete, and collected in different ways across surveys. Although the new estimators are still affected by sample design and effort [9], they have a novel theoretical basis, perform better in multiple aspects and have good potentials to further improve when compared to previous approaches [31]. Our work also shows that under realistic ecological conditions and commonly achieved sampling effort, no existing estimator can reliably estimate true richness and detect small richness differences across space or time when data are poor or spatially heterogeneous. These results come from considering both ecological and methodological uncertainties to a greater degree than previous works. They suggest that many previously detected biodiversity trends or lack thereof may have come from methodological and data deficiencies, and that investments in statistical research and sample design are critical for biodiversity monitoring [8–10]. Overall we found *Ω* to be a robust, general-purpose richness estimator.

The proposed *Ω* richness estimators for species richness achieve a superior balance of low bias, precision and accuracy in detecting richness differences compared to abundance-based asymptotic estimators including Chao1 [8], GP [34], ACE [36] and Jackknife1 [30] as shown by simulated communities containing imperfect local observations and spatial heterogeneities. *Ω* accounts for the mean and variance effects of patches sampled (sample size), fractions of individuals observed within each species (catchability), abundance (rarity), clustering (spatial variance) and occupancy, all readily computed from spatial abundance data and without the need to independently assess survey effectiveness. *Ω* harnesses information already present in common surveys, thus it is no more data-hungry than other methods we assessed. The proposed method can be used to study spatial and temporal trends [22] as well as their relationships with other environmental and biological covariates, without the confounding effects of these covariates on richness estimates, such as in some multi-species occupancy models [27,28].

*Ω* was the most accurate estimator across simulated scenarios. There remains a downward bias (increasing underestimation when true richness increases) to all richness estimators including *Ω*, with the exception of the incidence-based Chao2 [35] and Jackknife2 [30], which had the minimal bias when looking across all replicates despite ignoring abundances (they only relied on spatial occupancy). Incidence-based estimators were built to account for spatial and species catchability variations [32,35]. However, these estimators were the least precise and often the least accurate, meaning repeated observations will result in vastly different estimates. If absolute richness is the sole aim, these incidence-based estimators coupled with many independently repeated (data-hungry) surveys are recommended. The trade-off, in addition to imprecision, is the inability to resolve small differences between communities under conditions that mix imperfect local observation with spatial heterogeneity. By contrast, across the different scenarios that simulated datasets represent, *Ω* detected trends correctly more often than without correction (i.e. observed richness) and any other estimators under any spatially heterogeneous conditions while maintaining the lowest bias among abundance-based estimators. Results from empirical applications to the Barro Colorado Island tree census [54] and the British Columbia seaweed survey [39] largely agreed with the above conclusions. Although uncorrected observed richness tends to suggest empirical trends more often than estimators, observed richness does not consider the uncertainty from the observation process and thus may be over-confident in trend detection and direction as simulation results suggested. Finally, an improved bootstrapping procedure that accounts for spatial and species dependence of individual observations in a single survey showed that *Ω* was more precise than other estimators, especially relative to Chao2. High precision and low bias by themselves are not sufficient but necessary features for an accurate estimator. We recommend obtaining a variety of estimators when assessing community diversity and detecting change, with *Ω* being the best abundance-based estimator and overall best at detecting richness differences, *Ω*_0_ being overall best under low spatial heterogeneity conditions, and Chao2 being the least biased but requiring more replicated observations.

*Ω*-type estimators contain survivorship bias [42] because observed species tend to have higher abundance and higher occupancy than unobserved species. If survivorship bias can be corrected (as revealed by the Taylor expansion version *Ω_T_*)—and this may very well be possible by studying how spatial and abundance statistics change with subsampling and downsampling experiments—the proposed approach would come very close to being the perfect estimator (see the idealized *Ω_C_*). In addition, non-independence of spatial samples (*k*), such as when sampled patches are close together in space or time but counted as independent samples, adds downward bias to all *Ω* estimators by inflating inferred observation probabilities. This problem is apparent for the Barro Colorado Island census where subsampling led to insufficient correction by *Ω*, where *k* probably overestimated the number of independent spatial samples. The non-independence effect appears to outweigh the upward bias from *Ω*'s assumption of sampling individuals and patches with replacement when the actual sampling is without replacement—also a unique feature of the census. One way to address non-independence is relying on biological knowledge to demarcate independent patches, as we did for the British Columbia dataset by assuming that transects, not quadrats, are independent, or assuming that sets of quadrats belong to independent habitat types, for example from detailed analyses on Barro Colorado Island [54]. Alternatively, deriving an effective sample size may help correct downward biases from sample non-independence. Finally, insights from occupancy models that estimate observation probability from repeated sampling [29], when available, may be incorporated into *Ω* estimators in the future to harness sample dependence.

The detection of richness change has previously been addressed using rarefaction and extrapolation techniques that control for sampling effort [13,14], as opposed to uncontrolled asymptotic estimators (including *Ω*). Future research may apply rarefaction and extrapolation techniques to *Ω* and evaluate these performances in detection along with other estimators. Additionally, proper confidence bounds will help quantify detection uncertainty, which remains an outstanding issue for all richness comparison methods [23] even though our bootstrapping captures methodological uncertainties. Regardless, asymptotic estimators have the advantage of producing absolute rather than relative richness, which may be an indispensable biodiversity assessment criterion [4].

Our method is amenable to extensions. For example, spatial incidence data where abundance counts are unavailable can be easily handled. With local observable abundance *mn* fixed to a sufficiently high value for observed species (set to greater than 10 when preparing the data), the observation probability per occupied patch (equation (2.1)) is effectively 1, meaning that the only effect on *Ω* is equating the observation probability per patch *D_ix_* with occupancy *p_i_* (equation (2.2)). This simple extension illustrates the potential for the current approach to handle a variety of data qualities including surveys designed for biodiversity assessment, unstructured observational data and mixed data meta-analyses. We leave it for future research to quantify and improve the breadth and performance of *Ω*-type richness estimators across different data types.

We have offered an improved asymptotic richness estimator that already performs well in balancing bias, precision and accuracy, supporting *Ω* as today's standard. On the other hand, we identified many more open questions and improvement pathways that reveal the inherent difficulties in estimating richness and detecting change [16–21]. Ultimately, any richness estimator will be sensitive to whether the places and species we choose to observe represent the underlying community [55]. Thus, biological and field-methodological considerations along with estimation methods are indispensable for estimating richness and detecting changes.

## Data Availability

All code and data are provided on Zenodo [40], and an R-package *Richness* is available on Github at https://github.com/EWTekwa/Richness. The data are provided in the electronic supplementary material [56].
